# Navigating Esthetic Challenges: Immediate Implant Placement and Comprehensive Restorative Solutions—A Clinical Case With 2-Year Follow-Up Study

**DOI:** 10.1155/2024/1186299

**Published:** 2024-11-07

**Authors:** Gerardo Guzman-Perez, Carlos A. Jurado, Saad Alresayes, Franciele Floriani, Silvia Rojas-Rueda, Akimasa Tsujimoto

**Affiliations:** ^1^Periodontics Residency Program, CEMRO, Morelia, Mexico; ^2^Department of General Dentistry, Division of Operative Dentistry, The University of Tennessee Health Science Center, Tennesse, USA; ^3^Department of Prosthetic Dental Sciences, King Saud University College of Dentistry, Riyadh, Saudi Arabia; ^4^Department of Prosthodontics, The University of Iowa College of Dentistry and Dental Clinics, Iowa City, Iowa, USA; ^5^Division of Dental Biomaterials, University of Alabama at Birmingham School of Dentistry, Birmingham, Alabama 35233, USA; ^6^Department of Operative Dentistry, School of Dentistry, Aichi Gakuin University, Nagoya 464-8651, Japan; ^7^Department of Operative Dentistry, College of Dentistry, University of Iowa, Iowa City IA 52242, USA; ^8^Department of General Dentistry, School of Dentistry, Creighton University, Omaha NE 68102, USA

**Keywords:** crowns, esthetic dentistry, immediate implant, implantology, veneers

## Abstract

**Introduction:** The esthetics of immediate implant dentistry can be challenging. Clinical considerations for immediate implant placement have demonstrated long-term success rates comparable to traditional delayed implant protocols. However, it is a sensitive technique that requires proper treatment planning as well as meticulous execution to be predictable and successful in the long term.

**Methods:** This clinical situation demands extensive knowledge of soft and hard tissue management for atraumatic tooth extraction followed by implant therapy, along with an understanding of the available materials to meet esthetic needs. This case report features a female patient with the chief complaint of needing to replace an anterior crown due to loss of retention. After a comprehensive oral assessment and cone beam computed tomography (CBCT) radiographic examination, it was determined that the crown on Tooth #9 was fractured at the subgingival level. Furthermore, gingival zenith positions displayed differences at the keratinized mucosa level in Teeth #7, #8, and #9, and the metal marginal areas of the porcelain-fused-to-metal (PFM) crowns in the anterior area of Teeth #5, #7, #8, #9, and #10 were apparent. The procedure involved atraumatic extraction of Tooth #9, followed by immediate implant placement. Crowns on Teeth #5, #7, #8, and #10 were replaced, and veneers on Teeth #6 and #11 were fabricated using press lithium disilicate-reinforced ceramic. Connective tissue graft (CTG) was contoured before final implant restorations.

**Results:** The final implant crown was restored using a prefabricated abutment with a titanium base and lithium disilicate ceramic dental material. A well-planned combined treatment, including atraumatic tooth extractions for immediate implants and ideal contouring of soft tissues, can significantly impact the outcome of esthetic restorations.

**Conclusions:** Single immediate implant–supported crowns in the esthetic zone were able to fulfill the patient's esthetic expectations.

## 1. Introduction

Numerous important factors contribute to a favorable clinical outcome in immediate implant placement following extraction. These factors include the socket healing process, anatomical elements of the alveolar process (such as buccal bone width and the gap between the implant surface and the innermost part of the socket walls), the necessity for regenerative procedures, and enhanced surgical techniques [1]. Traditional implant installation methods often require a period of 12 months or more for bone regeneration after tooth extraction before placing a dental implant [2]. However, research on socket healing indicates that after 3–4 months, the socket is filled with newly created bone, allowing for the placement of a dental implant under favorable conditions to achieve primary stability. Esposito et al. reported in a randomized clinical trial no significant differences in failures, complications, or patient satisfaction on immediate, immediate-delayed (6 weeks), and delayed (4 months) postextraction and single implant placement [3].

Indeed, some publications have recommended methods for shortening treatment time in cases of failing dentitions by implanting implants into fresh extraction sockets. In 2010, Hurzeler et al. developed the socket shield technique (SST), providing a minimally invasive approach to preserving buccal bone and improving treatment outcomes with rapid implant placement [4–6]. In essence, the SST entails customizing the extraction tooth, separating the root mesiodistally into buccal and palatal fragments, and removing the palatal portion. [7, 8] Following that, the dental implant is placed, either engaging the buccal root section or leaving a gap in between. The gap can be grafted or left to heal naturally, depending on the surgical approach used [7–9].

Currently exists a tendency toward immediate and delayed implant placement following tooth extraction, with the objective of achieving effective and appropriate long-term results [10]. The impact of immediate and delayed implant placement on the esthetic performance of single maxillary anterior implants with intact bone walls and interproximal bone was studied in a one-year randomized clinical study, according to the findings of the study, both treatment modalities culminated in favorable esthetic outcomes [11].

Simultaneously, into the intricacies of the multidisciplinary approach, prosthodontics, specializing in the design and fabrication of dental prostheses, embraces a novel trajectory toward minimally invasive full-mouth rehabilitation. Combining veneers with tooth-supported crowns and a single immediate implant-supported crown in the esthetic zone represents a challenge for clinicians due to the risk of esthetic complications. Recently, monolithic and layered zirconia crowns have gained popularity for anterior crowns, dominating 84% of treatments in a material selection survey conducted by the National Dental Practice-Based Research Network [12]. These crowns were developed to provide high-strength restorations with an adequate clinical appearance [13]. Based on up to 7.5 years of clinical service in a survey of 188,695 restorations, it has been reported that layered zirconia single crowns had a higher fracture rate (2.83%) than lithium disilicate-reinforced glass-ceramic single crowns (0.96%) [14]. Therefore, clinicians should currently prefer lithium disilicate–reinforced ceramic crowns to single crowns. Unfortunately, limited case reports combining lithium disilicate–reinforced glass–ceramic veneers, tooth immediate implant placement, and immediate loading supported crowns in the esthetic zone of the same patient are available in the literature.

An electronic search involving immediate implant placement associated with connective tissue graft (CTG) in nonmolar areas was conducted in Pubmed (Table 1). The current article is divided into two parts: socket healing with immediate implant placement review, and case report. Therefore, this case report describes a conservative approach including atraumatic extraction of the maxillary central incisor and replacement with a single immediate implant placement in a fully guided surgery, and using lithium disilicate–reinforced ceramics in the esthetic zone to restore single immediate implant-supported crowns, and veneers in 2 years of follow-up.

### 1.1. Socket Healing With Immediate Implant Placement Technique

Implant placement immediately after tooth extraction is considered a preventive measure against or minimizer of dimensional reduction in the alveolar ridge [15, 16]. Several authors have recommended this approach to enhance esthetics and reduce the need for additional surgical interventions like ridge augmentation [17, 18]. However, the changes in soft and hard tissues and the esthetic outcomes around these implant restorations are not consistently favorable; therefore, the inclusion of regenerative procedures as part of tooth extraction and immediate implant procedures contributes to maintaining the contour of the alveolar bone [19].

The dimensional changes in the alveolar bone after immediate implant installation have been thoroughly investigated in various experimental studies [18–24]. In 2005, Araújo and Lindhe conducted the first study that initiated a comprehensive discussion on the fate of the socket walls and the timing of implant placement. Their conclusion highlighted that early implant placement depends on the level of the buccal bone wall during the initial phase of socket healing following implant installation [19].

In a prospective study with an 18-month follow-up, Tortamano et al. evaluated patients undergoing immediate implant placement and provisional prosthesis installation. The study's findings revealed no failures in implants or prostheses, and there was no occurrence of peri-implant soft tissue recession. The authors concluded that immediate implants associated with provisional restorations could serve as a predictable option for replacing missing teeth, especially when socket bone walls remain intact (Table 1) [21].

More recently, a systematic review and meta-analysis focused on the soft tissue outcomes of CTGs combined with immediate implant placement and restoration in the esthetic zone. The CTG in conjunction with immediate implant placement and restoration significantly enhanced the thickness of gingival tissues compared to cases without CTG. However, no significant differences were observed in the level of soft tissues around immediately placed implants, whether with or without CTG (Table 1) [22].

## 2. Materials and Methods

**Figure 1:**
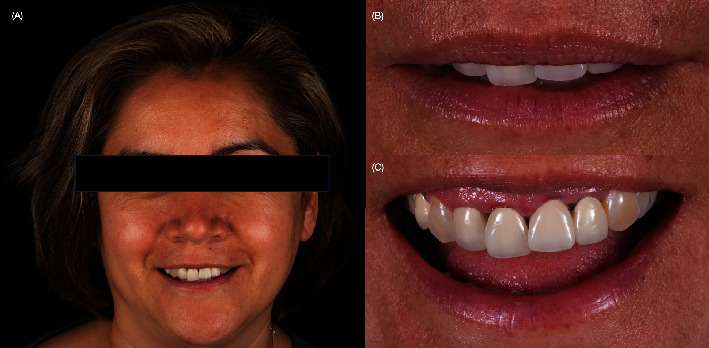
Extra-oral evaluation. (A) Initial extra-oral smiling; (B) smile at rest, and (C) smile with maximum display of teeth.

**Figure 2:**
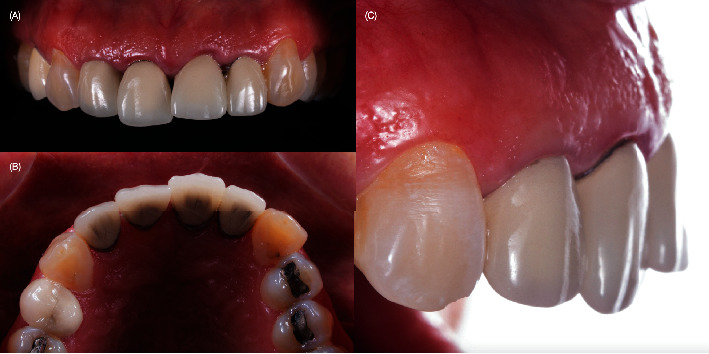
Intraoral evaluation. (A) Frontal view. (B) Incisal view. (C) Lateral view.

**Figure 3:**
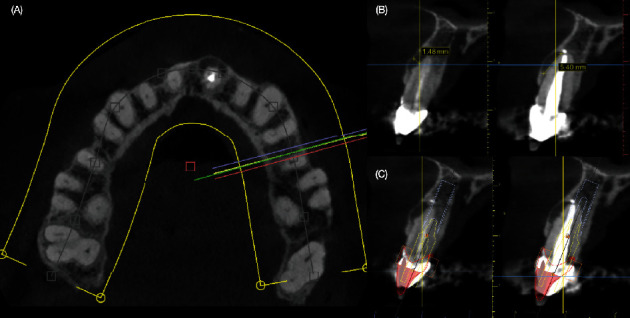
Cone beam computed tomography evaluation (CBCT). (A) Aligning the CBCT to see transversal views, (B) evaluation of the fractured tooth (crossing point of vertical and horizontal lines indicates root fracture), and (C) planning for the immediate implant placement.

**Figure 4:**
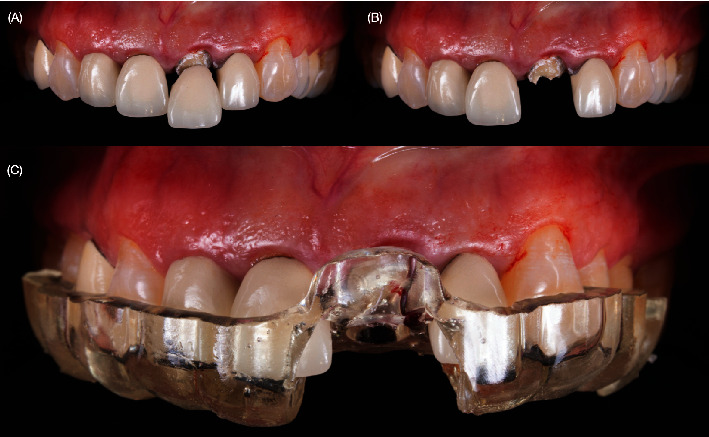
Crown removal and try-in of surgical guide. (A, B) Removing broken crown and (C) try-in of the surgical guide.

**Figure 5:**
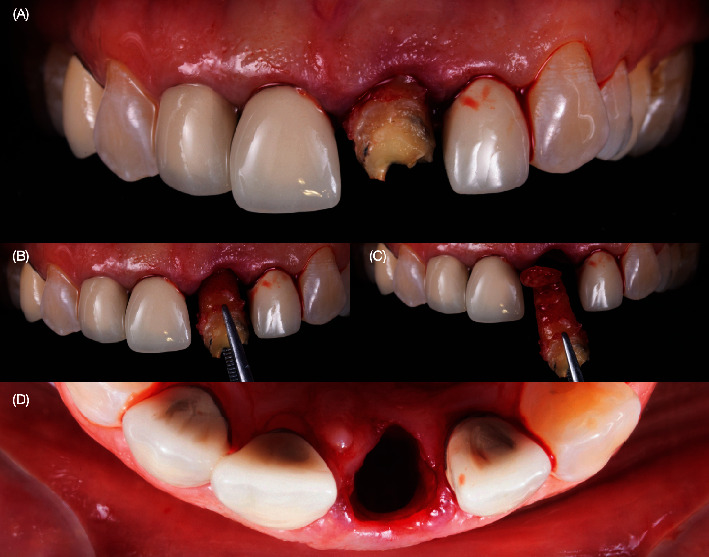
Atraumatic extraction. (A–C) Removing the tooth and (D) occlusal view of the socket maintaining the buccal plate.

**Figure 6:**
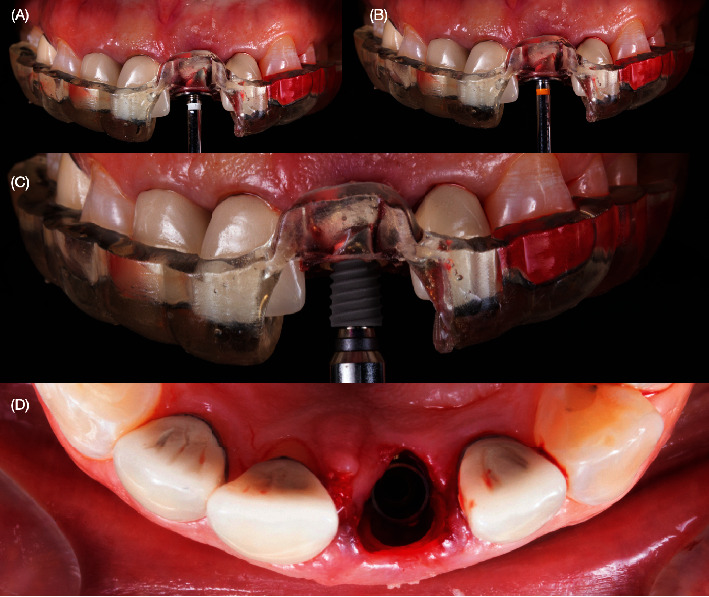
Implant placement. (A–C) Implant was placed. (D) Occlusal view of the socket after implant placement.

A 40-year-old female patient presented to the clinic with the chief complaint of the maxillary left central incisor crown loss of retention and disliking the metal marginal area of the porcelain-fused-to-metal (PFM) crowns in the anterior area (Figure 1). After a comprehensive oral assessment and CBCT radiography examinations, the patient was diagnosed with a maxillary left central incisor fractured at the subgingival level, uneven incisal edges for both central incisors, metal margin display at the gingival level for crowns on both central and lateral incisors, an uneven maxillary gingival margin, and incisal wear for both maxillary canines (Figures 2 and 3). The treatment options consisted of replacement of PFM crowns with lithium disilicate–reinforced glass–ceramic crowns for the right lateral, central incisor, and left lateral incisor. Additionally, veneers were proposed for the right first premolar and the right and left canines due to the patient's wide smile line and to achieve a shade match of all anterior teeth. Replacement of the upper left central incisor with a single immediate implant placement and immediate loading of single supported crown CTGs for buccal volume augmentation and to rectify the midfacial recession were also proposed. Planning for tooth extraction and an immediate implant placement was managed using CBCT scan radiography, and the thickness of buccal and lingual bone allowed the digital placement of an implant with 11.5 mm length and 4.0 mm diameter (ETIII NH, Hiossen, Englewood Cliffs, NJ, United States) (Figure 3). A surgical guide was digitally designed and printed out (Anycubic Photo, Anycubic, Shenzhen, China) of clear resin (Anycubic high Clear Resin, Anycubic) [25]. The surgical guide was tried and evaluated intraorally (Figure 4). Atraumatic flapless tooth extraction was conducted with piezoelectric instruments (Piezomed, W & H, Bürmoos, Austria) in order to preserve the intact buccal plate (Figure 5). The surgical guide was positioned, and an implant 11.5 mm × 4.0 mm (ETIII NH, Hiossen, Englewood Cliffs, NJ, United States) was inserted (Figure 6).

**Figure 7:**
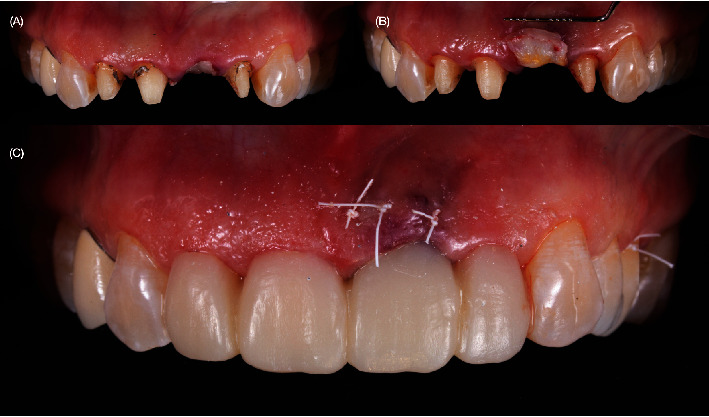
Crowns removal and provisional. (A) PFM crowns removed, (B) soft-tissue grafting procedure on the implant, and (C) tooth-supported provisional placement.

**Figure 8:**
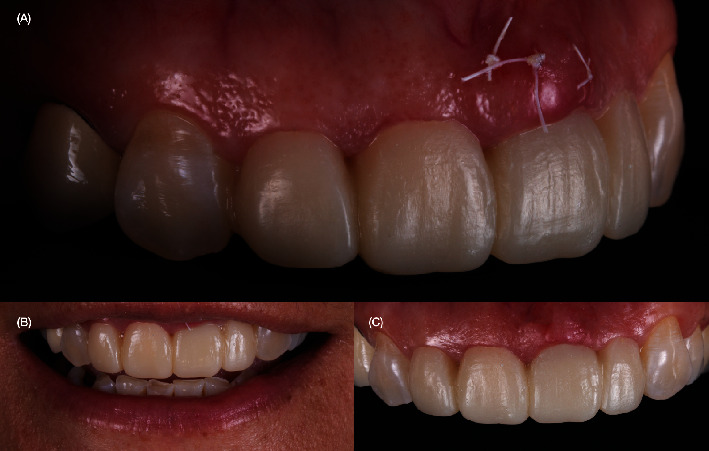
Healing time. (A) One month after implant placement. (B) Smile during healing process. (C) Three months healing time with screw-retained implant provisional restoration.

The PFM crowns for the upper maxillary right lateral, central incisors, and left lateral incisor were removed, and CTG, a 1-mm thick and 5-mm wide CTG, was harvested from the palate after local anesthesia with 40 mg/mL + 0.01 mg/mL of articaine. The length of the CTG was equal to that of the site to reconstruct measured from the distal to the mesial papilla. The harvested CTG was tunneled into a pouch made with a microblade between the labial mucosa and the cortical plate of the socket. Two horizontal mattress sutures in the mesial and distal aspects of the sockets were used to stabilize the CTG in position (Figure 7). An immediate interim restoration or a customized healing cap was used to help stabilize the blood clot protecting and covering the CTG. In cases in which a minor gingival recession was detected, the tunneled flap was coronally displaced with sling sutures to fully cover the CTG and prevent its necrosis. The healing process of the soft tissue was monitored, and at 4 months after implant placement, the provisional approach was modified to include single crowns and screw-retained provisional restoration (Figure 8).

**Figure 9:**
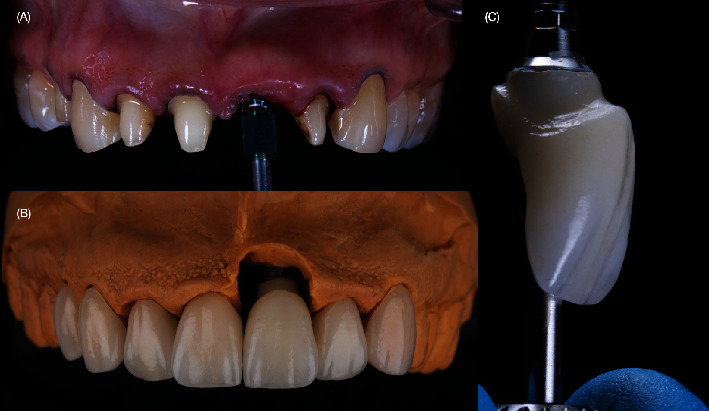
Fabrication of final restorations. (A) Final impression for teeth and implant. (B) Fabrication of tooth- and implant-supported restoration. (C) Screw-retained implant-supported crown with custom abutment and titanium base.

**Figure 10:**
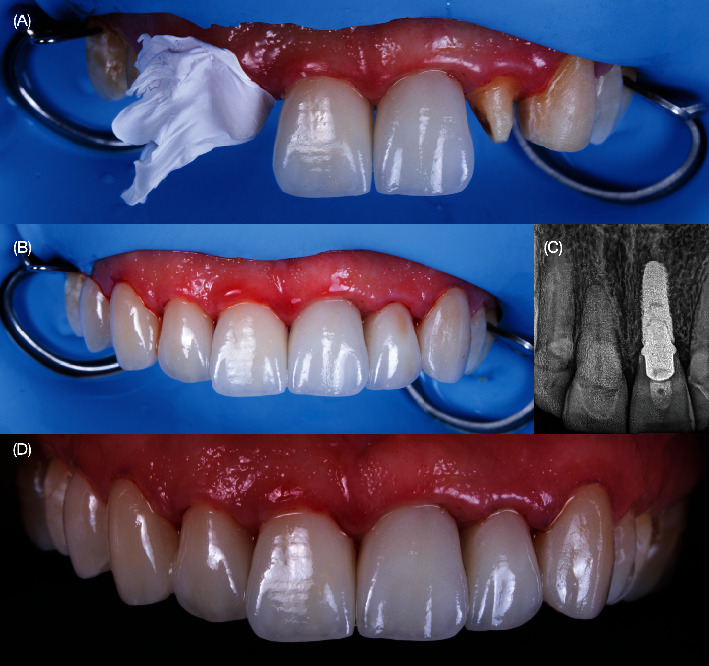
Placement of final restorations. (A) Placement of the rubber dam, cementation of the right central incisor crown, and screw retention of the implant crown. (B) Radiograph of the screw-retained implant crown. (C) Occlusal view of the final restorations. (D) Final frontal view of the restorations.

The final impression of the teeth and implant was taken. Subsequently, lithium disilicate–reinforced thin veneers for the upper maxillary left first premolar, left canine, and right canine were made, while full coverage lithium disilicate-reinforced crowns were created for the right lateral incisor, right central incisor, and left lateral incisor. Additionally, a screw–retained lithium disilicate implant–supported crown was fabricated (Figure 9). The tooth-supported restorations were pretreated before cementation, first with 4.9% hydrofluoric acid (IPS Porcelain Etch, Ivoclar Vivadent, Schaan, Liechtenstein) which was rinsed with water and air dried, and then treated with primer (Monobond Plus, Ivoclar Vivadent). On tooth surfaces, total etching was performed with 37% phosphoric acid (Total Etch, Ivoclar Vivadent), before they were rinsed, air dried, and primed (Primer A+B, Multilink Autolink, Ivoclar Vivadent) for 20 s. A rubber dam was placed, and to accommodate the implant, the dam was modified by being cut to allow access to the gingival surface prior to cementation. First, the crown for the maxillary left central incisor was cemented (Variolink Esthetic, Ivoclar Vivadent) accordingly to the manufacturer's instructions, and the implant crown was screwed in to evaluate the midline, followed by cementation of the crowns for the left lateral incisor and right lateral incisor and finally the veneers for the right first premolar and both maxillary canines (Figure 10).

**Figure 11:**
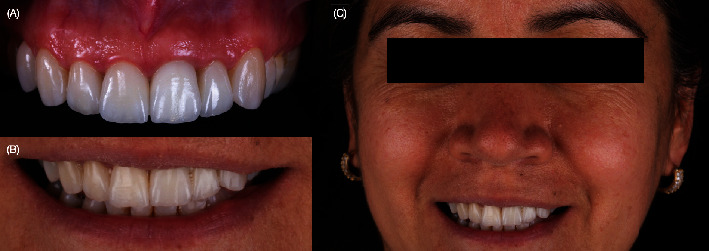
Two-year follow-up: (A) Front view showing the harmonious keratinized tissue between the implant and natural teeth and (B, C) facial front view smile.

The rubber dam was removed, and excess cement was removed with Blade #12 (Surgical Scalpel Blade no. 12, Salvin Dental Specialties, Charlotte, NC, United States). Occlusion maximum intercuspation, excursive movements, and protrusion were checked. The patient was pleased with the shade and shape of the final restorations. A full-mouth guard was provided to protect the restorations. At the 2-year follow-up, the restorations still matched the existing dentition harmoniously, and the patient was still satisfied (Figure 11).

## 3. Results

A multidisciplinary approach was integral to the treatment plan, considering factors such as buccal bone presence and tissue biotype to guide the decision between early or delayed implant placement. The plan also addressed esthetic concerns, including maintaining the interproximal papilla between the implant and natural teeth, distributing interproximal space between upper incisors, and achieving color-blending with different structures (tooth and implant).

Digital implant planning played a crucial role in ensuring accuracy in the 3D implant position. Emphasis was placed on implant depth to provide an adequate emergence profile and achieve gingival symmetry between the implant and natural teeth. Immediate implant placement was executed, and to prevent complications from both biological and esthetic perspectives, CTG**s** were employed to enhance tissue augmentation. Factors such as interproximal attachment of adjacent teeth, the presence of bone on the buccal surface of the implant, and horizontal and vertical implant positions were considered. Provisional restorations were pivotal for assessing esthetics and maintaining the interproximal papilla between the implant and natural teeth. Careful preservation of natural teeth through precise preparation allowed for the application of milled lithium disilicate reinforced glass-ceramic single crowns. These crowns exhibited robust adhesive properties with resin cement, ensuring a favorable long-term prognosis during the **2** years of follow-up (Figure 11).

## 4. Discussion

Achieving esthetic outcomes by combination of thin veneers, tooth-supported crowns, and single immediate implant–supported crowns in the esthetic zone poses a more intricate challenge compared to employing a single type of restoration. The patient's initial evaluation incorporated intra and extraoral photos, enabling the clinician to assess esthetic concerns related to the metal visibility around the PFM crowns, the mobility of the maxillary right central incisor crown, and offering guidance on tooth shade. Esthetic risk assessment must be carried out in consultation with the patient, leveraging diagnostic models, CBCT radiography, and intra and extraoral photos to optimize the planning of extraction and immediate implant placement. CBCT was used in the current report to evaluate the fractured maxillary left central incisor, assess the possibility of immediate implant placement, and design a printed surgical guide for the immediate implant. Digital planning for implant placement allows the fabrication of a surgical guide to place the implant in the desired position [25].

Traditional tooth extraction includes reflection of the mucoperiosteal flap followed by tooth evaluation against the surrounding bone structures by forced application with forceps [26, 27]. Atraumatic tooth extraction aims to reduce the trauma to the bone with forceps, elevators, luxators, or desmotomes during the extraction in order to reduce postoperative complications like loss of alveolar width and height [27]. This atraumatic tooth extraction was performed with desmotomes and only vertical forces, and this maintained the buccal plate which was critical for the following immediate implant placement.

Immediate implant placement after tooth extraction offers positive outcomes, including high survival rates and attractive esthetic results, appealing to both clinicians and patients. A systematic review assessing the survival and success rates of implants immediately placed after extraction reported a 98.4% success rate at 2 years, with marginal bone loss of less than 1 mm [28]. In a clinical trial comparing immediate and delayed implant placement, spanning 143 patients and 264 implants with a 5-year follow-up, no significant differences in failure rates were observed, with both approaches showing a success rate of 92.4% in the maxilla and 94.7% in the mandible [29]. The decision between immediate or delayed placement can be based on the treatment's impact on the patient, as long-term survival is generally unaffected. The patient in this case underwent immediate implant placement to save time and minimize surgical procedures. A systematic review and meta-analysis comparing the survival rate and peri-implant tissue changes in implants placed in fresh extraction sockets versus healed sockets indicated that immediate implant placement and loading in single-tooth edentulous spaces is a well-accepted treatment [30]. The CTG technique, designed to minimize peri-implant tissue architectural changes, contributes to successful functional and esthetic outcomes, as shown in a randomized clinical trial evaluating soft tissue augmentation during implant placement in patients with a thin gingival phenotype. The study demonstrated particularly good esthetics based on the pink esthetics score up to 12 months after permanent restoration [31].

Implant provisional restorations play a crucial role in achieving the ideal development of marginal mucosa and mesial and distal papilla contours [32]. In this case report, an immediate loading provisional restoration was applied during immediate implant placement, for adequate development of peri-implant subgingival contours (i.e., critical and subcritical contours), shaping the soft tissue until the desired gingival architecture was achieved before the final restoration. To develop an ideal emergence profile, a careful design and fabrication of the interim restoration is critical to the final treatment result [33]. Different factors, such as the vertical and horizontal position of the implant as well as peri-implant tissue architecture and characteristics, have to be taken into consideration, ascertaining its provision of a correct support or pressure to the gingiva [34]. A biologically driven subgingival restorative design is needed to optimize the architecture of the peri implant tissues and to maintain its stability over time [35]. Provisional restorations were instrumental in assessing and maintaining the interproximal papilla between the implant and natural teeth. A systematic review and meta-analysis assessed the prevalence of interproximal contact loss between implant restorations and adjacent teeth, considering factors such as age, gender, follow-up time, and arch location [36]. The prevalence of interproximal contact loss was high, particularly with the mesial contact. No significant differences were found concerning age, gender, or mandibular versus maxillary arch. Interproximal open contact between implant restorations and adjacent natural teeth emerged as a risk factor for peri-implant disease [36].

Lithium disilicate is a type of reinforced glass–ceramic material that can be used for many types of restorations in anterior and posterior regions [37, 38]. A retrospective study evaluating the survival rate of lithium disilicate crowns found that only 0.91% of restorations failed, and it concluded that this type of material has relatively low fracture rates [39]. Furthermore, a recent retrospective clinical study of 1075 lithium disilicate CAD/CAM veneers with feather-edge margins found a survival rate of 99.83% and it concluded that lithium disilicate veneers show good clinical performance in terms of survival, color matching, ceramic surface, margin discoloration and integrity [39]. Due to the proven success of lithium disilicate-reinforced ceramic restorations, the patient's existing crowns were removed and replaced with lithium disilicate crowns, and both canines and left first premolar teeth were minimally prepared for lithium disilicate thin veneers.

The optical characteristics of ceramic restorations are not only determined by the material itself but are also influenced by factors such as restoration thickness and the shade of the underlying material. In cases such as the present clinical case, involving the application of ceramics to diverse substrates, achieving a harmonious appearance requires meticulous attention. To ensure satisfactory color matching in this clinical case, different veneer thicknesses were applied to the canines and the right first premolar. However, the use of a prefabricated abutment with a white color for the implant restoration imposed limitations on achieving a seamless match. While opting for a zirconia abutment might improve esthetics, the presence of a thick gingival tissue phenotype, in this case, led us to consider the minor esthetic issues in the coronal area as acceptable.

Within the limitations of this clinical case report, a multidisciplinary approach was employed in the treatment plan to address biological, esthetics, and functional aspects to facilitate the oral functions and to enhance the patient's quality of life. It would be possible to treat teeth in this case individually, rather than doing everything at once. However, it was possible to minimize the number of surgical procedures and coordinate the treatment of each tooth to optimize esthetic outcomes. Thus, we decided to perform the treatment as described here.

## 5. Conclusions

Challenging clinical scenarios combining veneers, crowns, and a single immediate implant in the esthetic zone can be achieved, but thoughtful treatment planning taking into consideration dental, periodontal, and restorative needs is fundamental. Atraumatic tooth extractions for immediate implants and ideal contouring of the soft tissues can impact the outcome of the final result.

## Figures and Tables

**Table 1:** Clinical study characteristics of immediate implant placement associated with connective tissue graft (CTG) in nonmolar areas.

**Study**	**Design**	**Procedures**	**Teeth**	**Outcome Measurements**
Gómez-Meda et al.	Prospective study	Immediate implant and CTG	Nonmolar	Provide sufficient volume to peri-implant tissues in the vestibular area of anterior maxillary implants.
Ferrantino et al.	Prospective study	Implant placement with immediate nonfunctional provisional restoration in a fresh extraction socket	Nonmolar	CTG is not mandatory to achieve successful esthetic outcomes for a well-planned immediate implant placement with immediate nonfunctional provisional restoration in a fresh extraction socket.
Puisys et al.	Randomized clinical trial	Connective tissue graft (CTG) with immediate implant placement and provisionalisation or porcine-derived collagen matrix (PDCM) as grafting material	Nonmolar	Both treatment protocols resulted in comparable esthetic outcomes after 1 year of follow-up.
Athiel et al.	Systematic review	Evaluate the soft tissue outcomes of connective tissue graft combined with immediate implant placement and restoration in the esthetic zone	Esthetic zone	The use of CTG with immediate implant placement and provisional restoration can be considered to improve the soft tissue profile and minimize mucosal recession in the esthetic zone.
Cosyn et al.	Prospective study	Evaluate the preservation of pink esthetics in immediate implant technique with CTG	Esthetic zone	Preservation of pink esthetics is possible following immediate implant treatment. However, to achieve that, CTG may be necessary.

## Data Availability

Data used to support the findings of this study are available from the corresponding author upon request.
